# Loss of miR-24-3p promotes epithelial cell apoptosis and impairs the recovery from intestinal inflammation

**DOI:** 10.1038/s41419-021-04463-4

**Published:** 2021-12-18

**Authors:** Artin Soroosh, Kai Fang, Jill M. Hoffman, Ivy K. M. Law, Elizabeth Videlock, Zulfiqar A. Lokhandwala, Jonathan J. Zhao, Sepehr Hamidi, David M. Padua, Mark R. Frey, Charalabos Pothoulakis, Carl R. Rankin

**Affiliations:** 1 Vatche and Tamar Manoukian Division of Digestive Diseases, Department of Medicine, University of California Los Angeles, Los Angeles, CA USA; 2 Department of Pathology and Laboratory Medicine, University of California Los Angeles, Los Angeles, CA USA; 3 The Saban Research Institute, Children’s Hospital Los Angeles, Los Angeles, CA USA; 4 Department of Pediatrics and Department of Biochemistry and Molecular Medicine, University of Southern California Keck School of Medicine, Los Angeles, CA USA

**Keywords:** Apoptosis, Drug delivery

## Abstract

While apoptosis plays a significant role in intestinal homeostasis, it can also be pathogenic if overactive during recovery from inflammation. We recently reported that microRNA-24-3p (miR-24-3p) is elevated in the colonic epithelium of ulcerative colitis patients during active inflammation, and that it reduced apoptosis in vitro. However, its function during intestinal restitution following inflammation had not been examined. In this study, we tested the influence of miR-24-3p on mucosal repair by studying recovery from colitis in both novel miR-24-3p knockout and miR-24-3p-inhibited mice. We observed that knockout mice and mice treated with a miR-24-3p inhibitor had significantly worsened recovery based on weight loss, colon length, and double-blinded histological scoring. In vivo and in vitro analysis of miR-24-3p inhibition in colonic epithelial cells revealed that inhibition promotes apoptosis and increases levels of the pro-apoptotic protein BIM. Further experiments determined that silencing of BIM reversed the pro-apoptotic effects of miR-24-3p inhibition. Taken together, these data suggest that miR-24-3p restrains intestinal epithelial cell apoptosis by targeting BIM, and its loss of function is detrimental to epithelial restitution following intestinal inflammation.

## Introduction

Ulcerative colitis (UC) is a chronic inflammatory disease of the colon and rectum. For patients with UC, achieving and maintaining mucosal healing is an important predictor of sustained disease remission [1]. Following a flare of inflammation, mucosal repair requires intestinal epithelial cells to proliferate, migrate, and resist apoptosis [2–4]. Molecular pathways such as Wnt and EGFR drive proliferative events [5, 6] while integrins regulate migration into the wound [7, 8]. Apoptosis is orchestrated by families of proteins such as Bcl-2 that can be activated by either extrinsic or intrinsic apoptotic signals, initiating a cascade of events that result in Caspase activation, PARP cleavage, and DNA fragmentation [9].

Various studies over the past 15 years have profiled UC patient tissue for alterations in microRNA expression [10]. These experiments have uncovered a number of specific microRNAs with functional significance for the pathophysiology of UC. In a recent study, we reported that microRNA-24-3p (miR-24-3p) is expressed in the intestinal epithelium and is elevated in UC patients [11]. We also observed that overexpression of miR-24-3p reduces caspase activity in intestinal epithelial cells. Therefore, we hypothesized that genetically removing or inhibiting miR-24-3p would enhance epithelial cell death and reduce mucosal repair after inflammation.

In this study, we first challenged miR-24-3p knockout or miR-24-3p-inhibited mice with colitis. We observed that either method of miR-24-3p manipulation resulted in worse histological outcomes compared to controls. As miR-24-3p inhibition dramatically increased epithelial apoptosis and impaired recovery from colitis, we then investigated the molecular mechanism through which miR-24-3p regulates apoptosis. Our data support a model in which miR-24-3p suppresses epithelial apoptosis by targeting the pro-apoptotic Bcl-2 family member BIM. Together our data suggest that miR-24-3p is essential for mucosal protection and repair after an inflammatory insult to the colon.

## Materials and methods

### Creation of miR-24-3p knockout mice

CRISPR was used to generate both Mir24-1 and Mir24-2 knockout mice (Jackson Laboratory, Bar Harbor, ME, USA). Mir24-1 mice were crossed with Mir24-2 mice in house and 9-week-old female mice were used for colitis experiments as they could be co-housed with wild type (C56BL/6NJ) mice. For Mir24-1 the following gRNAs were used: 5′-CAGCTGATGCCACACGTGAT-3′, 5′-ACACCCCCACCCATCACGTG-3′, 5′-TGCCTCAGGCACTTACAGAT-3′ and the resulting mouse lacked Ch13: 63,448,871-63,449,119. The following genotyping primers were used for Mir24-1 knockout mice: 5′-CTGCCTCAAGGCTGTGTTGT-3′, 5′-TCTACAAATCCCCACCTCGG-3′. For Mir24-2 the following gRNAs were used: 5′-AGCTCAGTAGGCACGGGAGG, ATCAACTGTTTCAGCTCAGT-3′, 5′-TGAGCCTCCAGCAGACAACG-3′ and the resulting mouse lacked Chr8: 84,935,477-84,935,627. The following genotyping primers were used for Mir24-2 knockout mice: 5′-TATGTGAGACCCAGCCTGGT-3′, 5′-GAGGGGACATAACTGGCTTTT-3′.

### In vivo inhibitor treatment and colitis recovery model

Mouse-based experiments were done in accordance with the UCLA IACUC under protocol #2013-030. Mice were randomly assigned to each group. Custom large scale in vivo miRCURY locked nucleic acid (LNA) inhibitors were used (Qiagen, Hilden, Germany). These specific LNA inhibitors bind to the target miRNA, inhibiting RNA-Induced Silencing Complex loading. The catalog numbers for the control inhibitor and miR-24-3p inhibitor were 339203 YCI0201861-FZA and 339203 YCI0201383-FZA, respectively. The drugs were diluted in sterile 0.9% saline solution and 100 µl was injected per mouse at the concentrations stated in the figure legends. For intravenous (IV) injections, intraocular administration was performed. For colitis experiments, mice were injected intraperitoneally with 2.5 mg/kg of the inhibitor on days −1, 3, and 7 after dextran sulfate sodium (DSS). Male 9-week-old C57BL/6J mice were used (Jackson Laboratory). Mice were housed 4 per cage, maintained on a 12:12 h light-dark cycle, and given access to food and water ad libitum. To induce colitis, mice were given 5% DSS (w/v; MP Biomedical, Irvine, CA, USA) dissolved in drinking water. The DSS solution was refreshed on day 3. Mice received DSS for 5 days and were then switched to water alone and euthanized for tissue collection on day 10; this places tissue collection in the time frame when repair and recovery processes are active [12].

### Histology

After mice were euthanized via carbon dioxide and cervical dislocation, colons were dissected, flushed with phosphate buffered saline (PBS), cut open longitudinally and Swiss-rolled. After overnight fixation in 4% formaldehyde, colons were transferred to 70% ethanol. The Translational Pathology Core Laboratory at UCLA embedded them in paraffin, cut 5-micron sections, and performed hematoxylin and eosin (H&E) staining. An Aperio AT scanner was used to digitize the slides and ImageScope software was used to view the H&E slides (Leica Camera, Wetzlar, Germany). The double-blinded average histology score from three independent and concordant reviewers were based on immune cell infiltration and epithelial ulceration with the scores of 0–3 corresponding to none, mild, moderate, and severe, respectively.

### Ki67 Immunohistochemistry and scoring

A Bond Polymer Refine Detection kit was used for antigen retrieval, washes, and signal detection (Leica Biosystems, #DS9800). A Leica Bond RX machine was used to dewax and re-hydrate the slides. For antigen retrieval, slides were placed in buffer ER2 at 100 °C for 20 min. Slides were blocked in peroxide for 5 min. After blocking slides were washed three times. A Ki67 antibody was incubated with the sections for 1 h at the concentration of 1:1000 (Cell Signaling Technologies, #12202). Slides were then washed three times before being incubated with DakoCytomation Envision System Labelled Polymer HRP anti-rabbit for 10 min (Agilent, Santa Clara, CA, #K4003). Slides were washed three times for 2 min each. After polymer blocking, slides were washed five times in wash buffer then once in deionized water. Next, slides were incubated with Mixed DAB Refine for 10 min. After washing in deionized water wash three times, slides were incubated with hematoxylin for 10 min. Slides were then washed in wash buffer three times and once in deionized water. Finally, slides were dehydrated in series of alcohols, cleared with Histoclear and mounted with Permount (Vector Laboratories, Burlingame, CA). A blinded pathologist quantified the number of positive Ki67 positive epithelial cells per total epithelial cells within and surrounding the ulcer. Results are displayed as the average score of all ulcer associated regenerating crypts per mouse.

### RNA isolation and RT-PCR

A miRNeasy kit (Qiagen) was used to extract and purify miRNA from the mouse distal colon or cultured cells according to the manufacturer’s instructions. A LNA RT kit (Qiagen) was used to generate cDNA from miRNA, according to the manufacturer’s instructions. A CFX384 real-time PCR system was used to amplify and detect SYBR Green mediated signal (Bio-Rad Laboratories, Hercules, CA, USA). To measure miR-24-3p, a miRCURY LNA miRNA PCR assay primer was used (#YP00204260; Qiagen). The housekeeping primers were RNU1A1 and RNU5G (#YP00203909 and #YP00203908, respectively; Qiagen).

### Cell culture and transfections

Mycoplasma negative SW480 cells were purchased from American Type Culture Collection (Manassas, VA, USA) and were grown in DMEM + 10% FBS and 1% penicillin and streptomycin (Corning, Manassas, VA, USA). Non-transformed Young Adult Mouse Colon (YAMC) cells were grown under permissive conditions [33 °C in RPMI 1640 supplemented with 10% FBS, 5 units per mL Interferon-gamma (Peprotech, Cranbury NJ #315-05), 1% ITS (Corning, #25800CR), and 1% penicillin and streptomycin]. For experiments, cells were transferred to nonpermissive conditions [37 °C in transfection media without interferon or ITS]. To facilitate transfection, 70 μM lipofectamine RNAiMax was used according to the manufacturer’s instructions (Invitrogen, Carlsbad, CA, USA). For overexpression experiments, both a miR-24-3p mimic and a miRNA mimic negative control were used at a concentration of 50 nM (#PM10737 and #AM17110, respectively; Ambion, Austin, TX, USA). For inhibition experiments, the miR-24-3p antisense oligonucleotide and control inhibitor were used at a concentration of 50 nM. The control (#4390843) and BIM (#4390824-s195011) Silencer Select siRNAs were also used at 50 nM (Invitrogen). All transfections were performed in Optimem I (Invitrogen, #31985062).

### Western blotting

Semi-confluent cells were harvested in reducing Laemmlli sample buffer and subjected to 3 passes through a 25-gauge needle. Equal volumes of lysates were separated on 4–20% gradient denaturing polyacrylamide gels and transferred to PVDF membranes with a TransBlot Turbo RTA transfer kit (Bio-Rad). Membranes were blocked in 5% blotting-grade blocker in PBS with 0.05% Tween-20 and probed with antibodies against BIM (1:1000, Cell Signaling Technology, Danvers, MA, USA, 2933), Cleaved PARP (1:1000, Cell Signaling Technology, #9541), BCL-2 (1:1000, Cell Signaling Technology #2872), BAX (1:1000, Cell Signaling Technology, #5023) and Tubulin (1:5000, Sigma-Aldrich, St. Louis, MO, USA, #T5168). Horseradish peroxidase-labeled secondary antibodies were obtained from Jackson ImmunoResearch (West Grove, PA, USA). Clarity enhanced chemiluminescent reagent and a Chemidoc Touch (Bio-Rad) imaging system were used to develop and image the blots. All signal intensities reported are normalized to Tubulin.

### Apoptosis assays

*Caspase 3/7:* 100,000 cells were plated in transfection mix on 96 well plates. For staurosporine treatment, cells were transfected overnight before exposure to 2 µM staurosporine (Tocris Bioscience, Bristol, UK) or vehicle [dimethyl sulfoxide (DMSO) (1:1000)] for 4 h. For tumor necrosis factor (TNF) treatment, cells were transfected overnight before exposure to 100 ng/mL TNF (R&D Systems, #210TA020CF) or vehicle control (PBS). After treatment cells were then washed once in PBS, and 100 µl of a 1:1 mix of Caspase 3/7 GLO buffer to PBS was added per well (Promega, Madison, WI, USA). After 15–30 min of lysis, a Synergy HT plate reader was used to measure luminescence (BioTek Instruments, Winooski, VT, USA).

In vivo *TUNEL:* After routine deparaffinization and hydration, slides were microwaved in 10 mM citrate pH 6.0 supplemented with 0.05% Tween-20. A TUNEL (terminal deoxynucleotidyl transferase dUTP nick end labeling) kit was used to label apoptotic cells according to the manufacturer’s instructions (Roche, Basel, Switzerland). Nuclei were counterstained with DAPI and slides were mounted in Prolong Gold (Invitrogen). An Versa scanner was used to image the slides (Leica). TissueStudio software was used to automatically score and quantify the number of total and TUNEL-positive intestinal epithelial cells (Defiens Inc, Carlsbad, CA, USA).

In vitro *TUNEL:* 180,000 cells were plated in transfection mix on chambered slides. After 24 h, cells were treated with 2 µM staurosporine or DMSO vehicle for 4 h. Cells were then fixed with 4% formaldehyde and assessed for apoptosis with a Click-it TUNEL plus kit according to the manufacturer’s instructions (Invitrogen). Nuclei were counterstained with DAPI; slides were mounted in Prolong Gold (Invitrogen) and imaged on a Zeiss 710 confocal microscope (Carl Zeiss AG, Oberkochen, Germany). The cell counter function in ImageJ (National Institutes of Health, Bethesda, MD) was used to individually label and count cells.

### Lipocalin-2 assay

A mouse Lipocalin-2 DuoSet ELISA was used to measure fecal Lipocalin-2 (R&D Systems, #DY1857). One to two fresh pellets were weighed and diluted in 1 mL reagent diluent. 100 µL of the resulting sample was analyzed according to the manufacturer’s instructions. The positive control was feces from a DSS-treated mouse diluted 1:100 in reagent diluent.

### Statistical analysis

Statistical differences between two groups were evaluated using an unpaired *t*-test. For statistical analyses of more than two groups, a Tukey corrected, multiple comparisons, one-way ANOVA was performed. Sample sizes were based on prior experience with the same methods as a guide to what is needed to detect a difference. Graphing was completed using GraphPad Prism version 6 (GraphPad Software, Inc., San Diego, CA, USA). *P* < 0.05 was considered statistically significant.

## Results

### miR-24-3p knockout mice exhibit reduced mucosal repair after colitis

**Fig. 1:**
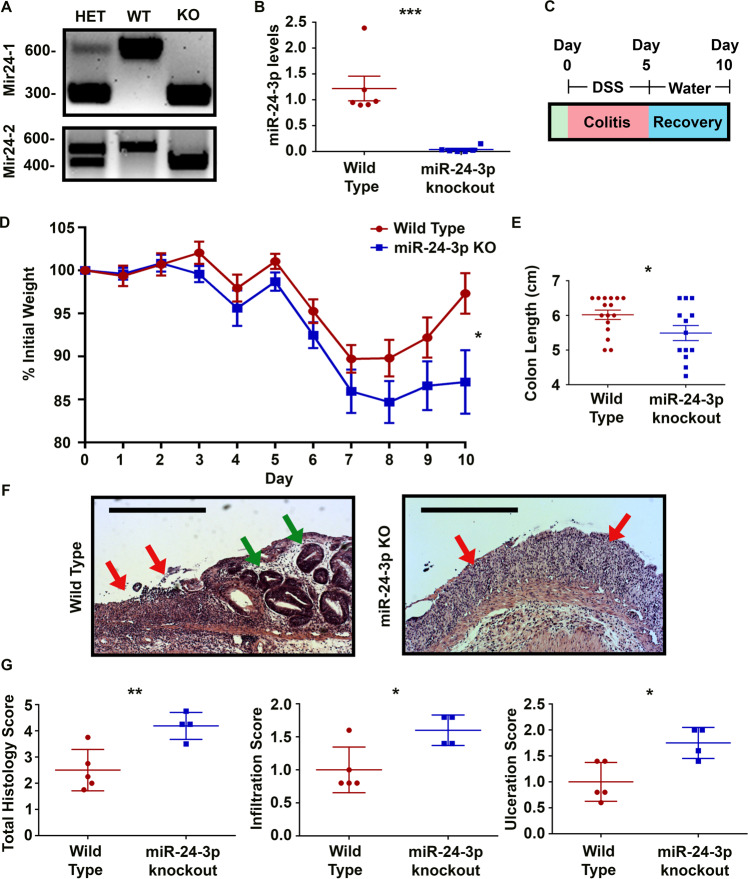
miR-24-3p knockout mice exhibit reduced mucosal repair after colitis. **A** Representative agarose gels of Mir24-1 and Mir24-2 genotyping. **B** After reverse transcription of colonic RNA, PCR was used to measure the level of miR-24-3p. *n* = 6 mice/group in two independent experiments. Mean ± SD. **C** A schematic describing the time course for colitis recovery experiments. **D** A graph of percent weight change over the course of recovery after colitis. *n* = 16/13 mice/group for wild type and miR-24-3p knockout, respectively, from three independent experiments. Mean ± SEM. **E** On day 10 of the protocol, mice were euthanized, and colon lengths were measured from the rectum to the cecum. *n* = 16/13 mice/group for wild type and miR-24-3p knockout, respectively, from three independent experiments. Mean ± SEM. **F** A representative image of the central colons of wild type or miR-24-3p knockout mice. Green arrows indicate areas of re-epithelization and red arrows indicate areas of ulceration. Scale bars = 0.5 mm. **G** Representative graphs of double-blind histology scores. *n* = 5/4 mice per group for wild type and miR-24-3p knockout, respectively, from two independent experiments. Mean ± SD. *p < 0.05; **p < 0.01; ***p < 0.001.

To determine whether miR-24-3p regulates the recovery from colitis we first created a knockout mouse. As miR-24-3p is produced at 2 separate genomic loci, Mir24-1 and Mir24-2, CRISPR was used to create a 248 base pair deletion surrounding Mir24-1 and a 150 base pair deletion surrounding Mir24-2 (Fig. 1A). When the levels of miR-24-3p were analyzed by qPCR in Mir24-1^−/−^ x Mir24-2^−/−^ compared to wild type mice, there was essentially a complete loss of miR-24-3p in the double knockout mice (miR-24-3p knockout). To determine if these mice had spontaneous colitis, we performed a Lipocalin-2 (Lcn-2) assay [13]. We did not observe any differences in fecal Lcn-2 between wild type and miR-24-3p knockout mice and neither appeared to have elevated Lcn-2 levels (Supplemental Fig. 1). A fecal pellet from a mouse with acute DSS colitis was used a positive control, and in line with other studies displayed a signal of over 2000 pg/mg Lcn-2 (Supplemental Fig. 1). Next, we subjected these mice to colitis. On day 0, mice received DSS, a molecule that induces colitis by causing distal colonic ulcerations [14]. Starting on day 5, mice were placed on water for an additional 5 days to enable the onset of mucosal repair mechanisms (Fig. 1C). We measured the weight of the mice each day as greater weight loss is indicative of worse disease [15]. When analyzing the percent weight change, a maximum weight loss for both wild type and miR-24-3p knockout mice occurred on day 7–8 (Fig. 1D), but the knockout mice lost considerably more weight (15–20% in knockout versus 10–15% in wild type mice). Furthermore, while by day 9–10 wild type mice had recovered nearly all lost weight, miR-24-3p knockout mice failed to regain weight. Another measure of colitis is reduced colon length. On day 10, miR-24-3p knockout mice displayed significantly shorter colons (Fig. 1E). After a double-blinded review of the entire colon histology, increased immune cell infiltrate and ulcerations to the epithelium were observed in knockout mice compared to wild type controls (Fig. 1F, G). While wild type mice started to recover the epithelium at the central/distal colon interface, the knockout mice still displayed only a layer of concentrated immune cells. These results suggest that genomic deletion of miR-24-3p impairs recovery from colitis.

### A cell-permeable miR-24-3p inhibitor is effective in the colon 5 days after one dose

**Fig. 2:**
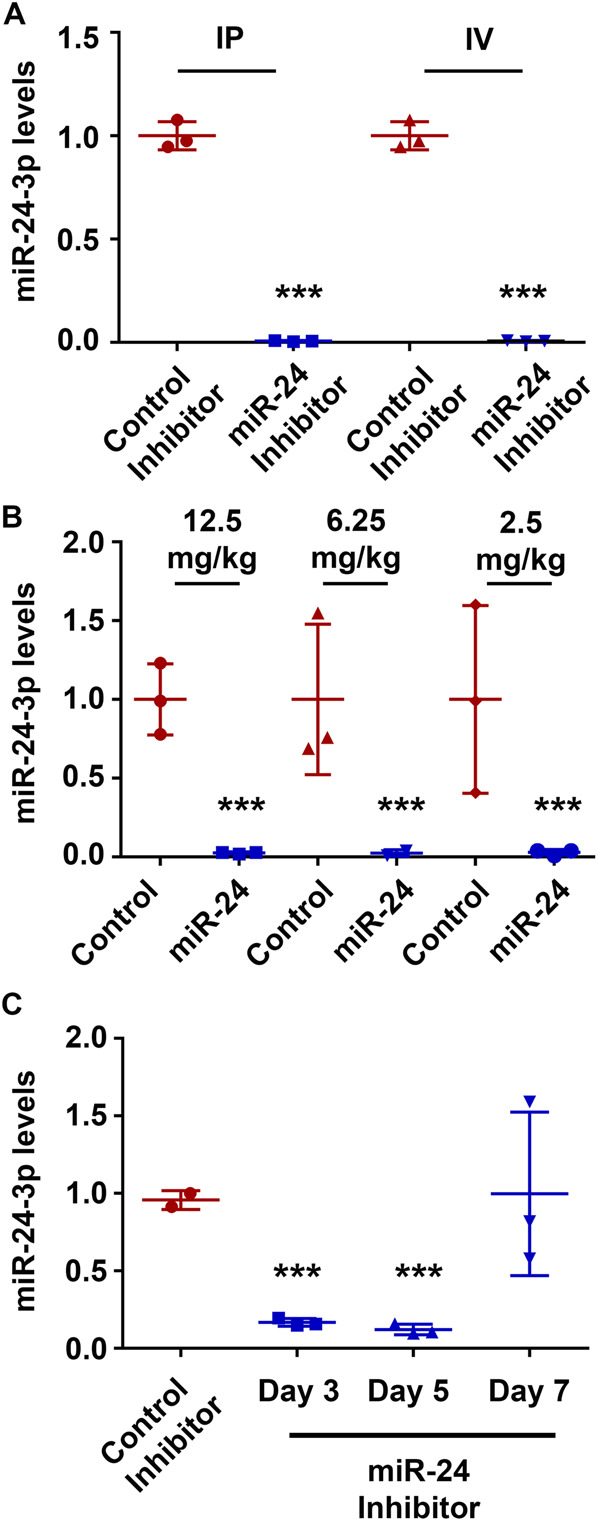
A cell-permeable miR-24-3p inhibitor is effective in the colon 5 days after one dose. **A** Mice received an intraperitoneal (IP) or intravenous (IV) injection of 12.5 mg/kg control inhibitor or miR-24-3p (miR-24) inhibitor and colonic RNA was extracted 4 days later. *n* = 3 mice per group, for IP/IV representative of two independent experiments. Mean ± SD. **B** IP injections of 12.5, 6.25, and 2.5 mg/kg of a miR-24-3p inhibitor (miR-24) were tested for their efficacy at 4 days post injection. *n* = 3 mice per group, representative of two independent experiments. Mean ± SD. **C** Mice were IP injected with 2.5 mg/kg of a miR-24-3p inhibitor and were sacrificed 3, 5, or 7 days later. *n* = 3 mice per group. Mean ± SD. ***p < 0.001.

We next optimized the use of a miR-24-3p inhibitor as a second means of testing this microRNA’s function. We first tested the route of administration and efficacy of a cell-permeable antisense oligonucleotide against miR-24-3p. We started with a high dose, 12.5 mg/kg, and analyzed its ability to inhibit miR-24-3p 3 days after either IV or intraperitoneal (IP) injections. Both methods of administration resulted in near complete loss of miR-24-3p detection by qPCR (Fig. 2A). As these LNA inhibitors function by binding and masking/sequestering the target miRNA [16], the loss in qPCR signal reflects the unavailability of miR-24-3p primers to bind to their target. We next determined that an IP injection with a dose as low as 2.5 mg/kg resulted in full inhibition; furthermore, at that dose, the effect persisted for 5 days but not 7 (Fig. 2B, C). From these experiments, we concluded that a 4-day, 2.5 mg/kg dosing interval would be sufficient to inhibit miR-24-3p expression in our in vivo DSS recovery model.

### Inhibition of miR-24-3p diminishes mucosal repair after colitis

**Fig. 3:**
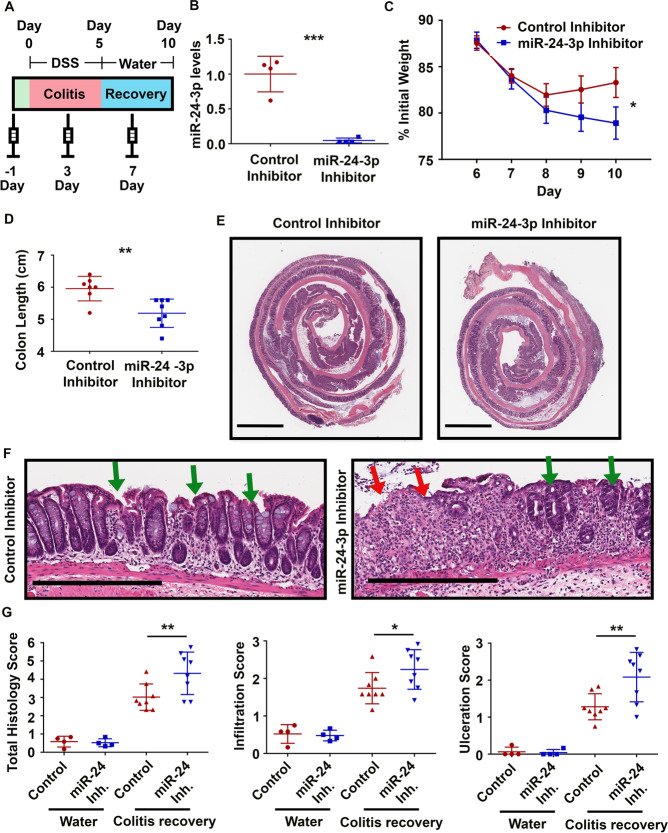
Inhibition of miR-24-3p diminishes mucosal repair after colitis. **A** A schematic describing the time course for inhibitor injections as it pertains to the colitis recovery experiments. **B** After colitis and recovery, the level of miR-24-3p in the distal colon was measured by RT-qPCR. *n* = 4 mice/group. Mean ± SD. **C** A graph of percent weight change over the course of recovery after colitis. *n* = 24 mice/group from three independent experiments. Mean ± SEM. Three independent experiments. **D** On day 10 of the protocol, mice were euthanized, and colon lengths were measured from the rectum to the cecum. *n* = 8 mice per from three independent experiments. Mean ± SD. **E** Representative H&E-stained Swiss-rolls used for histology scoring. Scale bars = 2 mm. **F** Magnified images of the central colons from control inhibitor or miR-24-3p inhibitor-treated mice. The green arrows indicate areas of re-epithelization and red arrows indicate areas of immune cell infiltration and epithelial ulceration. Scale bars = 0.4 mm. **G** Representative graphs of double-blind histology scores. *n* = 8 mice per group for water treated, *n* = 16 mice per group for DSS treated from two independent experiments. Mean ± SD. *p < 0.05; **p < 0.01; ***p < 0.001.

To test the effects of miR-24-3p inhibition on colitis, we injected mice on day −1, day 3, and day 7 with either a control inhibitor or the miR-24-3p inhibitor (Fig. 3A). On day 0, mice received DSS, which was withdrawn on day 5; mice were then followed for an additional 5 days to enable the onset of mucosal repair mechanisms. We confirmed near complete inhibition of miR-24-3p at the end of our 10-day protocol (Fig. 3B). As expected, mice treated with either the control inhibitor or the miR-24-3p inhibitor reached peak weight loss levels by day 8 (Fig. 3C). However, after peak weight loss, control inhibitor-treated mice began to recover whereas miR-24-3p-inhibited mice continued to lose weight. This resulted in a significant 8% difference in body weight between the two groups on day 10 (Fig. 3C). Furthermore, miR-24-3p-inhibited colons were almost a full centimeter shorter than control inhibitor-treated colons (Fig. 3D). To analyze histological damage, we H&E-stained Swiss-rolled colons. In control inhibitor-treated mice only the most distal region of the colon was ulcerated on day 10; however, miR-24-3p-inhibited mice were significantly ulcerated throughout the colon (Fig. 3E). When observing magnified segments of the central-distal region of the colons, crypt regeneration was apparent in control inhibitor-treated mice on day 10 (Fig. 3F). However, there was little crypt regeneration in miR-24-3p-inhibited mice (Fig. 3F). These effects were quantified by double-blinded reviewers who scored the extent and severity of immune cell infiltrate and epithelial ulceration, with the sum of the two being the total histology score. Histological scores were significantly elevated in the miR-24-3p inhibitor-treated DSS recovery group as compared to controls, confirming impaired intestinal restitution (Fig. 3G). We did not observe any differences in the histology of miR-24-3p inhibitor-treated mice without DSS treatment as compared to controls (Fig. 3F). The effects of miR-24-3p do not appear to be due to alteration in initial injury or inflammation, since histological scoring performed on day 5 (Supplemental Fig. 2A) showed no differences in ulceration or infiltration scores between control and miR-24-3p-inhibited mice (Supplemental Fig. 2B). Together, these results suggest that miR-24-3p inhibition weakens the recovery from acute colitis, and indicate that the effects in the knockout mice are not due to off-target effects of the gene editing.

### Inhibition of miR-24-3p enhances intestinal epithelial cell death after colitis

**Fig. 4:**
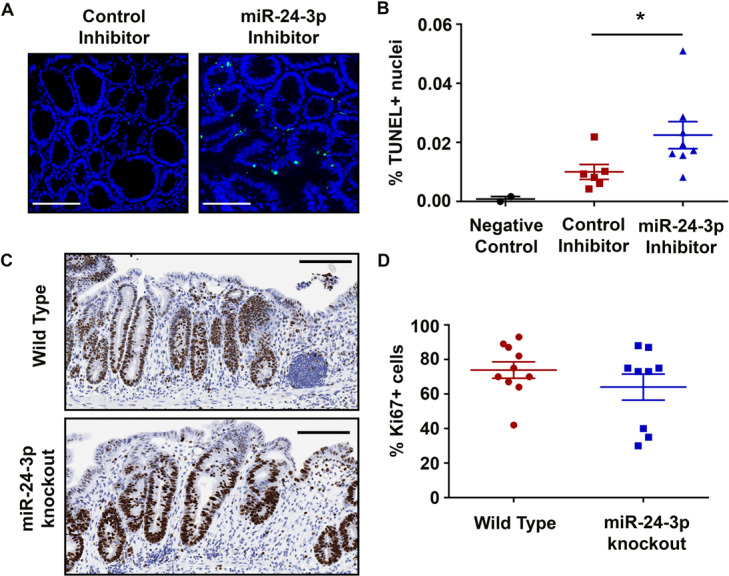
Inhibition of miR-24-3p enhances intestinal epithelial cell apoptosis during the recovery from colitis. **A**, **B** After TUNEL labeling of Swiss rolls, the central and distal epithelium were annotated with software and the total number of TUNEL-positive nuclei (green) were quantified and compared to the total number of nuclei (blue). Scale bars = 100 µm. *n* = 4 mice for control inhibitor, *n* = 6 mice for miR-24-3p inhibitor from two independent experiments. Mean ± SD. *p < 0.05. **C** Representative Ki67 immunohistochemistry images of epithelial cells surrounding the ulcerated epithelium. Scale bars = 150 µm. **D** A graph representing a pathologist’s assessment of Ki67 positive cells surrounding the ulcer. *n* = 10 for wild type mice, *n* = 9 for miR-24-3p knockout mice from two independent experiments. Mean ± SD.

To investigate the cause of attenuated recovery following colitis, we next analyzed whether miR-24-3p inhibition was causing increased intestinal epithelial cell apoptosis. Using a terminal UTP nucleotide end labeling (TUNEL) assay, we stained mouse colons harvested on day 10 of DSS recovery for DNA damage. Mice treated with the control inhibitor had few TUNEL-positive cells (Fig. 4A), suggesting low levels of apoptosis during epithelial restitution. However, miR-24-3p-inhibited mice had patches of TUNEL-positive cells throughout the epithelium. Automated quantification of these stained slides showed consistently elevated TUNEL-positive cells in miR-24-3p-inhibited mice compared to controls across multiple experiments (Fig. 4B). Another possible cause for the lack of epithelialization in mice lacking or with inhibited miR-24-3p could be stunting of epithelial cell proliferation post injury. To determine if proliferation was altered at day 10, we performed immunohistochemistry for Ki67 in miR-24-3p knockout and wild type mice. There was a high level of proliferation in colonocytes from both knockout and wild type mice (Fig. 4C). When quantified, there were no differences between the two genotypes (Fig. 4D). Taken together, these data suggest that miR-24-3p inhibition caused elevated apoptosis, which is a likely explanation for impaired recovery from colitis.

### miR-24-3p inhibition promotes Caspase 3/7 activation in vitro

**Fig. 5:**
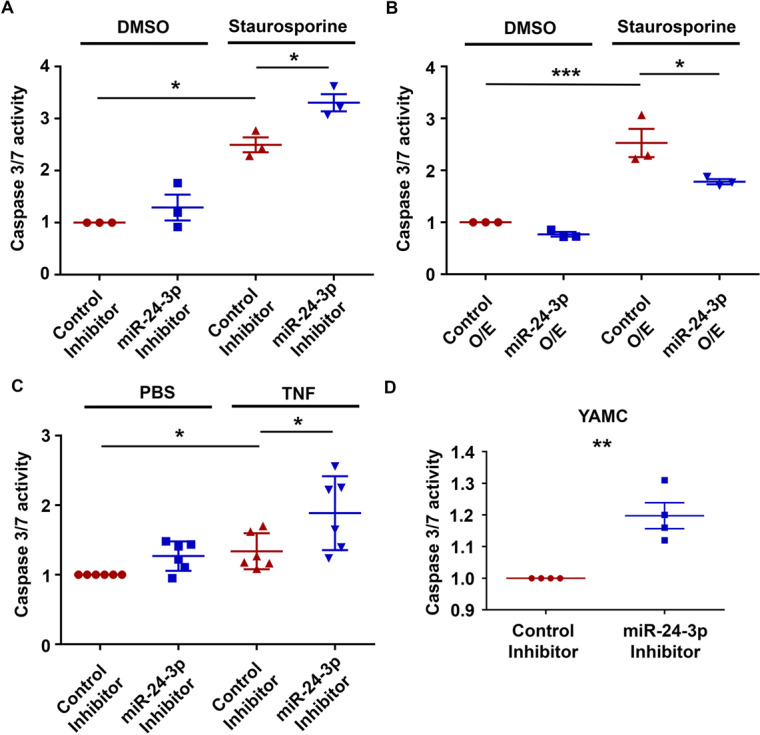
miR-24-3p inhibition promotes Caspase 3/7 activation in vitro. **A** SW480 intestinal epithelial cells transfected with either control or miR-24-3p inhibitor in the presence or absence of the cell death inducer staurosporine for 4 h were lysed in a buffer containing a luminescent caspase 3/7 substrate. Three independent experiments. Mean ± SEM. **B** SW480 cells transfected with either control mimic or miR-24-3p mimic (O/E) in the presence or absence of staurosporine for 4 h were lysed in a buffer containing a caspase 3/7 reactive luminescent buffer. Three independent experiments. Mean ± SEM. **C** SW480 cells transfected with either control or miR-24-3p inhibitor in the presence or absence of tumor necrosis factor (TNF) for 4 h were lysed in a buffer containing a luminescent caspase 3/7 substrate. Six independent experiments. Mean ± SEM. **D** YAMC (Young Adult Mouse Colon) non-transformed colonic epithelial cells were transfected overnight with a miR-24-3p inhibitor and caspase activity was measured 24 h later. Four independent experiments. Mean ± SEM. *p < 0.05; **p < 0.01; ***p < 0.001.

To determine if the increased epithelial cell death after miR-24-3p inhibition was due to immune cell-independent effects, we transitioned to in vitro studies using cultured epithelial cells. We conducted apoptosis assays on intestinal epithelial cells treated with either the miR-24-3p inhibitor or a miR-24-3p mimic, in the presence or absence of the cell death inducers staurosporine or TNF. We chose two apoptosis assays targeting different steps in the apoptotic cascade a Caspase 3/7 activity assay, an intermediary event, and TUNEL staining, which measures DNA fragmentation, a final event. Cells treated with the miR-24-3p inhibitor had increased caspase activity at baseline compared to control inhibitor-treated cells (Fig. 5A). Increased Caspase activity was also observed in cells treated with staurosporine plus the miR-24-3p inhibitor compared to cells treated with staurosporine plus the control inhibitor. Conversely, miR-24-3p overexpression also reduced Caspase activity induced by staurosporine (Fig. 5B). To determine if miR-24-3p inhibition can have additive effects on cell death for a more IBD relevant cell death inducer we treated SW480 cells with TNF. We observed that after 4 h of TNF treatment, Caspase activity was elevated in control inhibitor-treated cells (Fig. 5C). When comparing TNF-treated control inhibitor and miR-24-3p treated cells we observed that, similar to staurosporine, miR-24-3p inhibition elevated Caspase activity in the presence of TNF compared to controls. To determine if the effects of miR-24-3p inhibition on apoptosis were also observable in non-transformed intestinal epithelial cells we employed the YAMC cell line [17]. We observed that in the absence of any cell death inducer, Caspase activity was elevated when miR-24-3p was inhibited in YAMC cells (Fig. 5D). In summary, miR-24-3p expression promotes intestinal epithelial cell survival under proinflammatory and pro-apoptotic conditions.

### miR-24-3p promotes intestinal epithelial cell survival

**Fig. 6:**
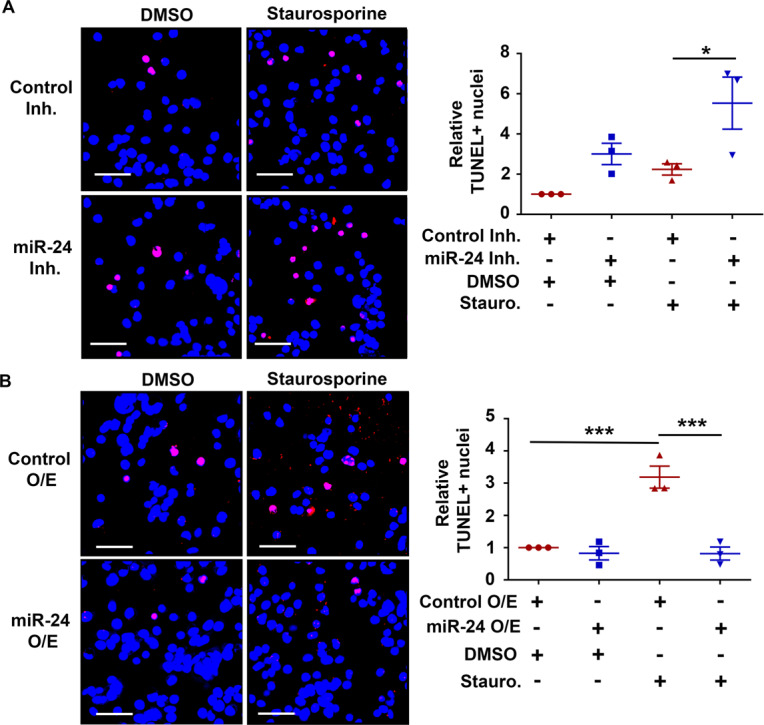
miR-24-3p promotes intestinal epithelial cell survival. **A**, **B** SW480 cells transfected with either inhibitors or mimics in the presence or absence of staurosporine were fixed and TUNEL stained. Magenta cells are TUNEL-positive and non-TUNEL-positive cell nuclei are labeled in blue. Three 250 × 250 µm fields were selected for quantification and are depicted in the graph. Three independent experiments. Mean ± SEM. Scale bars = 50 µm. *p < 0.05; ***p < 0.001.

To determine if the differences in caspase activation due to miR-24-3p manipulation result in alterations in DNA fragmentation TUNEL assays were performed. We observed that miR-24-3p inhibition increased the proportion of TUNEL-positive cells by ~2-fold (Fig. 6A). Furthermore, miR-24-3p inhibitor-treated cells co-treated with staurosporine had a 2-fold increase in TUNEL positivity compared to control inhibitor cells given staurosporine. While overexpression of miR-24-3p at baseline resulted in no significant effects on TUNEL staining, it repressed TUNEL positivity induced by staurosporine (Fig. 6B). These results suggest that miR-24-3p restrains intestinal epithelial cell death, likely explaining weakened recovery after DSS colitis when mice were treated with the miR-24-3p inhibitor.

### miR-24-3p regulates the pro-apoptotic protein BIM in intestinal epithelial cells

**Fig. 7:**
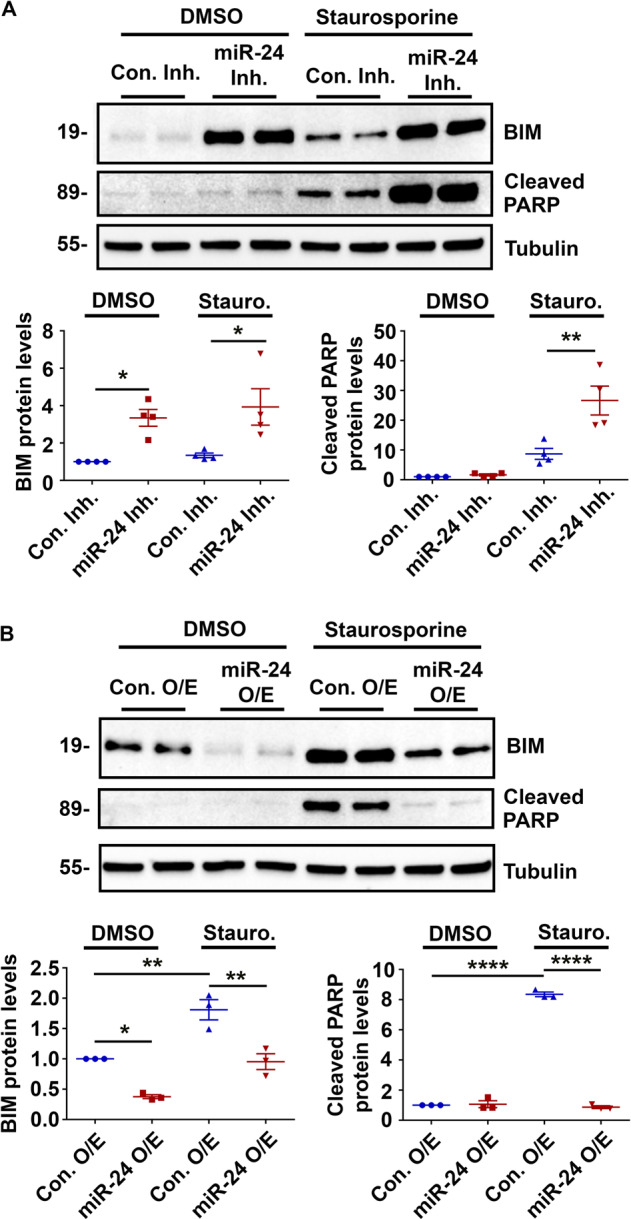
miR-24-3p regulates the pro-apoptotic protein BIM in intestinal epithelial cells. **A** Western blots and densitometric analysis of BIM, cleaved PARP and Tubulin from SW480 cells treated with control or the miR-24-3p inhibitor in the presence or absence of staurosporine. Four independent experiments. Mean ± SEM. **B** Western blots and densitometric analysis of BIM, cleaved PARP and Tubulin from cells treated with control or miR-24-3p mimic in the presence or absence of staurosporine. Three independent experiments. Mean ± SEM. *p < 0.05; **p < 0.01; ****p < 0.0001.

When comparing the seed sequence of miR-24-3p to the 3’UTR of the Bcl-2 family of apoptosis-associated proteins, BIM (Bcl-2 interacting mediator of cell death), a pro-apoptotic mediator, has perfect complementarity [18]. We therefore tested if miR-24-3p regulates BIM in intestinal epithelial cells. Western blots against BIM and cleaved PARP (Poly ADP-ribose polymerase), a downstream target of BIM, demonstrated that miR-24-3p inhibition alone increases BIM and cleaved PARP levels compared to cells treated with the control inhibitor (Fig. 7A). In staurosporine-treated cells, miR-24-3p inhibition also increased the levels of both BIM and cleaved PARP compared to control inhibitor-treated cells. When miR-24-3p was overexpressed, BIM levels dramatically decreased compared to control cells (Fig. 7B). Additionally, miR-24-3p overexpression was able to almost entirely revert the increases in BIM and cleaved PARP induced by staurosporine, demonstrating the potent anti-apoptotic abilities of a miR-24-3p analog in intestinal epithelial cells. To determine the specificity of the effects of miR-24-3p on BIM we analyzed two other BCL-2 family members, BCL-2 and BAX, by western blot. We did not observe either BCL-2 or BAX protein levels to be altered by either miR-24-3p manipulation or staurosporine treatment (Supplemental Fig. 3A–D).

### Downregulation of BIM reduces the induction of apoptosis caused by miR-24-3p inhibition

**Fig. 8:**
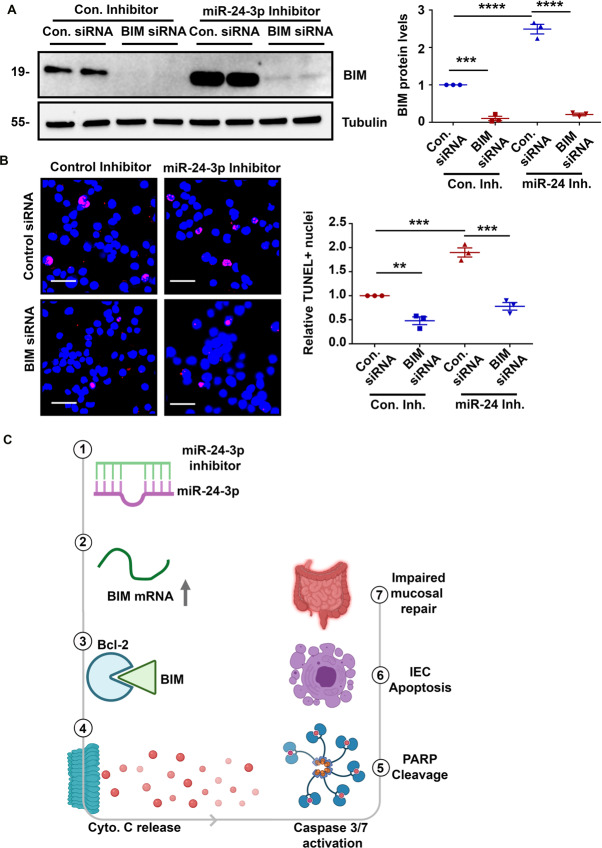
Downregulation of BIM reduces the induction of apoptosis caused by miR-24-3p inhibition. **A** Western blots and densitometric analysis of BIM and Tubulin from SW480 cells treated with staurosporine and either control or BIM siRNA plus either control inhibitor or miR-24-3p inhibitor. Three independent experiments. Mean ± SEM. **B** A TUNEL assay was used to measure the proportion of apoptotic cells. Magenta cells are TUNEL-positive and non-TUNEL-positive cell nuclei are labeled in blue. Three 250 × 250 µm fields were selected for quantification and are depicted in the graph. Three independent experiments. Mean ± SEM. Scale bars = 50 µm. **p < 0.01; ***p < 0.001; ****p < 0.0001. **C** We observed that when miR-24**-**3p was inhibited or removed from the genome (1) the mRNA and protein levels of BIM increase (2). BIM then stabilizes Bcl-2 (3) enabling Bax-mediated Cytochrome C release from the mitochondria (4). Cytochrome C activates caspases which result in events such as PARP cleavage (5). The activated pro-apoptotic enzymes then induce apoptosis in the intestinal epithelium (6). Induction of apoptosis during mucosal repair after colitis worsens outcomes (7).

In order to determine if the pro-apoptotic effects of miR-24-3p depletion require BIM, we used next used an siRNA to downregulate BIM. Transfection with anti-BIM siRNA resulted in a robust downregulation of BIM protein (Fig. 8A). BIM siRNA reduced TUNEL positivity rates with miR-24-3p inhibition to a level similar to control inhibitor plus control siRNA treatment (Fig. 8B). These experiments demonstrate that BIM contributes to the effects of miR-24-3p inhibition on apoptosis and is a likely player in miR-24-3p-mediated regulation of mucosal repair after colitis (Fig. 8C).

## Discussion

miR-24-3p is consistently upregulated in IBD [11, 19–21]. Genes that are upregulated in UC often are involved in mitigation of tissue injury and restoration of homeostasis. For example, the ErbB4 receptor tyrosine kinase and the non-coding RNA BC012900 are both elevated in UC but inhibition of either worsens disease and increases IEC death in vivo and in vitro [22, 23]. Localizing to the intestinal epithelium, miR-24-3p is likely to influence IEC homeostasis. One study from our lab has tried to ascribe a functional importance for miR-24-3p as it relates to IECs in vitro [11]. While in vitro studies are important to understanding cell type specific functions, colitis is a complex disease involving the interaction of many cell types. We therefore sought to determine if inhibition or loss of miR-24-3p would positively or negatively impact mice challenged with colitis. Overall, the findings presented here support a role for miR-24-3p in recovery from colitis through inhibition of apoptosis.

Both transient inhibition and knockout of miR-24-3p resulted in increased severity of colitis including a greater degree of weight loss and increased histologic ulceration inflammatory cell infiltration. The difference in body weight with knockout/inhibition of miR-24-3p was seen only following withdrawal of DSS suggesting that loss of miR-24-3p impaired mucosal in the recovery phase. This is supported by similar histologic scores at the completion of the colitis phase (day 5). Importantly, miR-24-3p knockout mice do not develop spontaneous colitis and have baseline bodyweight and levels of fecal lipocalin that are similar to wild type.

miR-24 has been shown to be involved in both promoting epithelial cell proliferation and inhibiting apoptosis [18, 24–29]. While knockout/inhibition of miR-24-3p increased apoptosis during recovery, there was no difference in epithelial proliferation at this time point. This suggests a role for regulation of apoptosis by miR-24-3p during healing from colitis. To determine whether there was an IEC-specific effect on apoptosis, we tested the effects of manipulating miR-24-3p in cultured colonic epithelial cells and observed similar increases in cell death upon inhibition of miR-24-3p. It appears that regardless of cell type miR-24-3p represses apoptosis.

We decided to focus on the pro-apoptotic protein BIM as multiple other studies have demonstrated that miR-24-3p directly binds to the 3’UTR of BIM, downregulating BIM protein levels and repressing apoptosis [18, 24]. It was evident that inhibition of miR-24-3p elevated BIM protein levels in cultured IECs even without a cell death inducer. Additionally, it appears that BIM was the sole Bcl-2 family protein regulated by miR-24-3p inhibition.

Our findings are in line with those of other studies showing that miR-24-3p directly targets many mRNAs associated with anti-apoptotic, anti-proliferation and DNA damage responses. Nearly every study demonstrates the same end result—that miR-24-3p improves cell survival. One other study has analyzed the effects of miR-24-3p on IECs in vitro [30]. They demonstrated that miR-24-3p overexpression reduced the pro-survival effects of PMS1 homolog 2 mismatch repair system component pseudogene 2 (PMS2L2). While different than the overall effects we see of miR-24-3p on survival these effects could be specific to DNA repair or PSM2L2 alone.

This study is not without limitations. First, our primary analysis represents a single snapshot during the disease time course. Analysis of histology between days 6 and 10 would provide more definitive understanding of whether the role of miR-24-3p is restricted to recovery, whether anti-apoptotic effects may be observed at earlier timepoints and whether there are changes in proliferation at other time points. Second, while we focused on BCL-2 family proteins, miR-24-3p has also been shown to target mRNAs of other pro-apoptotic proteins such as FAF1 (FAS-associated factor 1) and the senescence-associated protein p16 [31, 32]. Furthermore, miR-24-3p has been shown to regulate the apoptosis-inducing DNA damage response and extrinsic apoptosis pathway [25], both of which could also mediate the impaired healing after loss or inhibition of miR-24-3p [33, 34]. Finally, the presence/absence and function of miR-24 in immune cells also needs to be clarified in order to fully understand our results. It is clear that global inhibition of apoptosis worsens colitis as BIM global knockout mice do worse on colitis protocols [35]. It is likely that these mice do worse due to the fact that activated immune cells fail to die and keep producing inflammatory factors. As we have observed that miR-24-3p colonic expression might be specific to the epithelium, the possibility of cell-specific targeting of BIM through miR-24-3p could be key to future therapies. Regardless of the cause, it is apparent that global inhibition or loss of miR-24-3p results in worse inflammatory outcomes.

Current drug treatments for UC mainly target the immune system and inflammatory cytokines [36]. However, it is well noted that the best clinical sign for remission from inflammation is the re-epithelialization of the intestinal mucosa [1, 3]. Two major mechanisms for this restitution lie in the survival and proliferation of the epithelial cells. Our results suggest that miR-24-3p analogs could potentially enhance epithelial cell survival without negatively influencing proliferation, enabling the epithelium to withstand the apoptosis-inducing effects of the proinflammatory milieu. To address this, in vivo studies over-expressing miR-24-3p need to be performed. While it is currently technically difficult to overexpress microRNAs in vivo, future studies aimed at improving the stability and uptake of microRNA mimics, perhaps using lipid nanoparticles, could overcome these obstacles [37]. While miR-24-3p overexpression in vivo will likely improve colitis outcomes, the possibility of promoting colorectal cancer is apparent as many studies observe miR-24-3p to be elevated in cancer [38]. However, since the severity and duration of active inflammation correlate with elevated cancer risk in UC it is entirely possible that transient miR-24-3p based treatments could improve colitis outcomes without inducing cancer.

## Supplementary information

checklist

Author approval to changes

Supplemental Figures and Legends

## Data Availability

The materials described in this manuscript, including all relevant raw data, will be freely available to any researcher wishing to use them for non-commercial purposes.
