# Correction: Loss of miR-24-3p promotes epithelial cell apoptosis and impairs the recovery from intestinal inflammation

**DOI:** 10.1038/s41419-022-04559-5

**Published:** 2022-02-10

**Authors:** Artin Soroosh, Kai Fang, Jill M. Hoffman, Ivy K. M. Law, Elizabeth Videlock, Zulfiqar A. Lokhandwala, Jonathan J. Zhao, Sepehr Hamidi, David M. Padua, Mark R. Frey, Charalabos Pothoulakis, Carl R. Rankin

**Affiliations:** 1 Vatche and Tamar Manoukian Division of Digestive Diseases, Department of Medicine, University of California Los Angeles, Los Angeles, CA USA; 2 Department of Pathology and Laboratory Medicine, University of California Los Angeles, Los Angeles, CA USA; 3 The Saban Research Institute, Children’s Hospital Los Angeles, Los Angeles, CA USA; 4 Department of Pediatrics and Department of Biochemistry and Molecular Medicine, University of Southern California Keck School of Medicine, Los Angeles, CA USA

**Keywords:** Apoptosis, Drug delivery

Correction to: C*ell Death & Disease* 10.1038/s41419-021-04463-4, published online 18 December 2021

The original version of this article unfortunately contained a mistake. In the original version of this article, some data in Fig. 6 were presented incorrectly. The online version of this figure has been updated with the correct data. All the results previously reported as statistically significant remain so. There is no associated change to the text of the article. The authors regret this error.

**Fig. 6:**
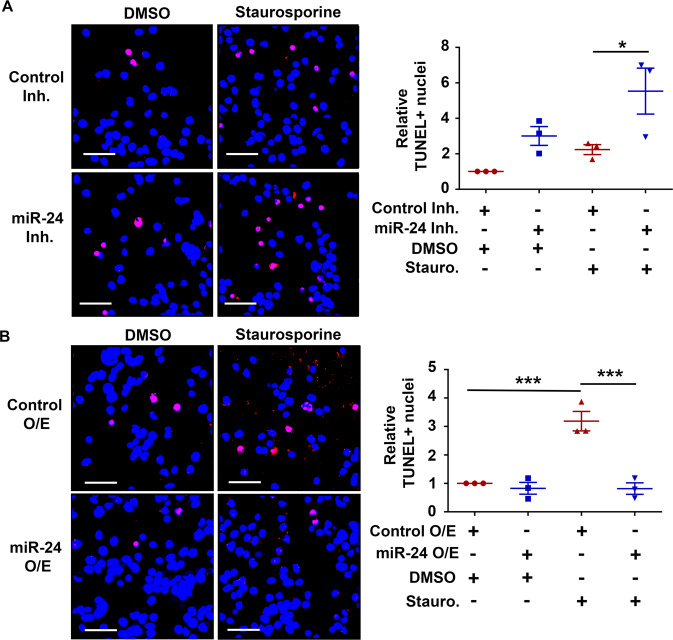
▓.

