# Supplementary material for: Loss of miR-24-3p promotes epithelial cell apoptosis and impairs the recovery from intestinal inflammation
Source: Cell Death Dis. 2021 Dec 18;13(1):8. doi: 10.1038/s41419-021-04463-4 (PMC8684555; doi:10.1038/s41419-021-04463-4)
Supplement: Supplementary file 2 — Author approval to changes [file 41419_2021_4463_MOESM2_ESM.pdf]

1. Artin Soroosh

Re: FW: article approval

ARTIN SOROOSH <asoroosh@ucla.edu>
Tue 11/30/2021 9:51 AM
To: Rankin, Carl <CRankin@mednet.ucla.edu>

I agree with the changes.

On Tue, Nov 30, 2021, 9:40 AM Rankin, Carl <CRankin@mednet.ucla.edu> wrote:

> Dear Artin, Harry, Jill and Mark
>
> I sent this to you over the weekend but included the figures so it was a really large email and Artin didn't get it. I wanted to send the manuscript alone to get your approval as the figures have not changed. See below remark from the journal.
>
> Thank you
>
> Rob
>
> **From:** Rankin, Carl <CRankin@mednet.ucla.edu>
> **Date:** Saturday, November 27, 2021 at 9:22 AM
> **To:** ARTIN SOROOSH <asoroosh@ucla.edu>, Fang, Kai <KFang@mednet.ucla.edu>, jillmayhoffman@gmail.com <jillmayhoffman@gmail.com>, KaMan.Law@kp.org <KaMan.Law@kp.org>, Videlock, Elizabeth J. <EVidelock@mednet.ucla.edu>, ali.lok97@gmail.com <ali.lok97@gmail.com>, Jonathan Zhao <jonathan.zhao2000@gmail.com>, Hamidi, Sepehr <SHamidi@mednet.ucla.edu>, David Padua <davepadua@gmail.com>, Frey, Mark <mfrey@chla.usc.edu>, Harry Pothoulakis <hpothoulakis@yahoo.com>
> **Subject:** article approval
>
> Dear Authors,
>
> One condition from the journal is that I compile a document containing email-based approvals from each author. The only difference between this version and the revision version is the addition of author contribution and data availability statements.
>
> Here is their exact wording:
>
> "Please request agreement from all authors including additions and deletions, these can be collected in the following way:

2. Kai Fang

RE: article approval

Fang, Kai <KFang@mednet.ucla.edu>
Mon 11/29/2021 9:17 AM
To: Rankin, Carl <CRankin@mednet.ucla.edu>

Dear Rob:

I agree the changes in the revised manuscript. Let me know if further information is needed.

Best regards.

Kai Fang

From: Rankin, Carl
Sent: Saturday, November 27, 2021 9:22 AM
To: ARTIN SOROOSH <asoroosh@ucla.edu>; Fang, Kai <KFang@mednet.ucla.edu>; jillmayhoffman@gmail.com <jillmayhoffman@gmail.com>; KaMan.Law@kp.org <KaMan.Law@kp.org>; Videlock, Elizabeth J. <EVidelock@mednet.ucla.edu>; ali.lok97@gmail.com <ali.lok97@gmail.com>; Jonathan Zhao <jonathan.zhao2000@gmail.com>; Hamidi, Sepehr <SHamidi@mednet.ucla.edu>; David Padua <davepadua@gmail.com>; Frey, Mark <mfrey@chla.usc.edu>; Harry Pothoulakis <hpothoulakis@yahoo.com>
Subject: article approval

Dear Authors,

One condition from the journal is that I compile a document containing email-based approvals from each author. The only difference between this version and the revision version is the addition of author contribution and data availability statements.

Here is their exact wording:

"Please request agreement from all authors including additions and deletions, these can be collected in the following way:

Email your co-authors with the change, and ask them to reply to your email confirming that they agree to these changes. Once you have collected these replies, please combine all of the co-authors' email responses in one document and upload this file to your submission."

Thank you,

Rob

3. Jill Hoffman

Re: article approval

Jill Hoffman <jillmayhoffman@gmail.com>
Tue 11/30/2021 10:17 AM
To: Rankin, Carl <CRankin@mednet.ucla.edu>

CAUTION - EXTERNAL EMAIL: Do not click links or open attachments unless you recognize the sender.

Dear Rob,
Thanks for sending along the most recent version of the paper. I have noted the additions made to the original manuscript and agree.
Thank you for the opportunity to be included in this work.
Best wishes,
Jill Hoffman

> On Nov 30, 2021, at 09:28, Rankin, Carl <CRankin@mednet.ucla.edu> wrote:
>
> Dear Artin, Harry, Jill and Mark
>
> I sent this to you over the weekend but included the figures so it was a really large email and Artin didn't get it. I wanted to send the manuscript alone to get your approval as the figures have not changed. See below remark from the journal.
>
> Thank you
>
> Rob
>
> **From:** Rankin, Carl <CRankin@mednet.ucla.edu>
> **Date:** Saturday, November 27, 2021 at 9:22 AM
> **To:** ARTIN SOROOSH <asoroosh@ucla.edu>, Fang, Kai <KFang@mednet.ucla.edu>, jillmayhoffman@gmail.com <jillmayhoffman@gmail.com>, KaMan.Law@kp.org <KaMan.Law@kp.org>, Videlock, Elizabeth J. <EVidelock@mednet.ucla.edu>, ali.lok97@gmail.com <ali.lok97@gmail.com>, Jonathan Zhao <jonathan.zhao2000@gmail.com>, Hamidi, Sepehr <SHamidi@mednet.ucla.edu>, David Padua <davepadua@gmail.com>, Frey, Mark <mfrey@chla.usc.edu>, Harry Pothoulakis <hpothoulakis@yahoo.com>
> **Subject:** article approval
>
> Dear Authors,
>
> One condition from the journal is that I compile a document containing email-based approvals from each author. The only difference between this version and the revision version is the addition of author contribution and data availability statements.
>
> Here is their exact wording:
>
> "Please request agreement from all authors including additions and deletions, these can be collected in the following way:
>
> Email your co-authors with the change, and ask them to reply to your email confirming

4. Ivy Law

Re: article approval

Ivy Law <ivylawkm@gmail.com>
Sat 11/27/2021 2:47 PM
To: Rankin, Carl <CRankin@mednet.ucla.edu>

CAUTION - EXTERNAL EMAIL: Do not click links or open attachments unless you recognize the sender.

Dear Robert,

Thanks for your email.

I agree to all changes made to this manuscript.

Kind regards,
Ivy

On Sat, Nov 27, 2021, 9:29 AM Rankin, Carl <CRankin@mednet.ucla.edu> wrote:

> Dear Ivy,
>
> One condition from the journal is that I compile a document containing email-based approvals from each author. The only difference between this version and the revision version is the addition of author contribution and data availability statements.
>
> Here is their exact wording:
>
> "Please request agreement from all authors including additions and deletions, these can be collected in the following way:
>
> Email your co-authors with the change, and ask them to reply to your email confirming that they agree to these changes. Once you have collected these replies, please combine all of the co-authors' email responses in one document and upload this file to your submission."
>
> Thank you,
>
> Rob
>
> UCLA HEALTH SCIENCES IMPORTANT WARNING: This email (and any attachments) is only intended for the use of the person or entity to which it is addressed, and may contain information that is privileged and confidential. You, the recipient, are obligated to maintain it in a safe, secure and confidential manner. Unauthorized redisclosure or failure to maintain confidentiality may subject you to federal and state penalties. If you are not the intended recipient, please immediately notify us by return email, and delete this message from your computer.

5. Beth Videlock

Re: article approval

Videlock, Elizabeth J. <EVidelock@mednet.ucla.edu>
Mon 11/29/2021 9:06 AM
To: Rankin, Carl <CRankin@mednet.ucla.edu>

I agree to the changes you have made.

Elizabeth J Videlock, M.D., Ph.D.
Assistant Professor

Vatche and Tamar Manoukian Division of Digestive Diseases
David Geffen School of Medicine at UCLA
MRL 2736
Office Phone: 424-327-4473
Email: evidelock@mednet.ucla.edu

https://www.uclahealth.org/gastro/research/videlock-lab
@DrVidelock

From: Rankin, Carl <CRankin@mednet.ucla.edu>
Sent: Saturday, November 27, 2021 9:22 AM
To: ARTIN SOROOSH <asoroosh@ucla.edu>; Fang, Kai <KFang@mednet.ucla.edu>; jillmayhoffman@gmail.com <jillmayhoffman@gmail.com>; KaMan.Law@kp.org <KaMan.Law@kp.org>; Videlock, Elizabeth J. <EVidelock@mednet.ucla.edu>; ali.lok97@gmail.com <ali.lok97@gmail.com>; Jonathan Zhao <jonathan.zhao2000@gmail.com>; Hamidi, Sepehr <SHamidi@mednet.ucla.edu>; David Padua <davepadua@gmail.com>; Frey, Mark <mfrey@chla.usc.edu>; Harry Pothoulakis <hpothoulakis@yahoo.com>
Subject: article approval

Dear Authors,

One condition from the journal is that I compile a document containing email-based approvals from each author. The only difference between this version and the revision version is the addition of author contribution and data availability statements.

Here is their exact wording:

"Please request agreement from all authors including additions and deletions, these can be collected in the following way:

Email your co-authors with the change, and ask them to reply to your email confirming that they agree to these changes. Once you have collected these replies, please combine all of the co-authors' email responses in one document and upload this file to your submission."

Thank you,

Rob

6. Ali Lokhandwala

Re: article approval

Zulfiqar Ali Lokhandwala <ali.lok97@gmail.com>
Sat 11/27/2021 9:26 AM
To: Rankin, Carl <CRankin@mednet.ucla.edu>

CAUTION - EXTERNAL EMAIL: Do not click links or open attachments unless you recognize the sender.

Hi Robert,
I agree to these changes on this manuscript, thank you for reaching out and best regards

Zulfiqar Ali Lokhandwala

On Sat, Nov 27, 2021 at 9:23 AM Rankin, Carl <CRankin@mednet.ucla.edu> wrote:

> Dear Authors,
>
> One condition from the journal is that I compile a document containing email-based approvals from each author. The only difference between this version and the revision version is the addition of author contribution and data availability statements.
>
> Here is their exact wording:
>
> "Please request agreement from all authors including additions and deletions, these can be collected in the following way:
>
> Email your co-authors with the change, and ask them to reply to your email confirming that they agree to these changes. Once you have collected these replies, please combine all of the co-authors' email responses in one document and upload this file to your submission."
>
> Thank you,
>
> Rob
>
> UCLA HEALTH SCIENCES IMPORTANT WARNING: This email (and any attachments) is only intended for the use of the person or entity to which it is addressed, and may contain information that is privileged and confidential. You, the recipient, are obligated to maintain it in a safe, secure and confidential manner. Unauthorized redisclosure or failure to maintain confidentiality may subject you to federal and state penalties. If you are not the intended recipient, please immediately notify us by return email, and delete this message from your computer.

--
Zulfiqar Ali Lokhandwala (he/him)
MD/MPH Candidate, Class of 2026
Oregon Health and Science University, School of Medicine
OHSU-PSU, School of Public Health
lokhandw@ohsu.edu I 559.302.8288

7. Jon Zhao

Re: article approval

Jonathan Zhao <jonathan.zhao2000@gmail.com>
Sun 11/28/2021 5:11 PM
To: Rankin, Carl <CRankin@mednet.ucla.edu>

CAUTION - EXTERNAL EMAIL: Do not click links or open attachments unless you recognize the sender.

I agree to these changes.

On Sat, Nov 27, 2021 at 9:23 AM Rankin, Carl <CRankin@mednet.ucla.edu> wrote:

> Dear Authors,
>
> One condition from the journal is that I compile a document containing email-based approvals from each author. The only difference between this version and the revision version is the addition of author contribution and data availability statements.
>
> Here is their exact wording:
>
> "Please request agreement from all authors including additions and deletions, these can be collected in the following way:
>
> Email your co-authors with the change, and ask them to reply to your email confirming that they agree to these changes. Once you have collected these replies, please combine all of the co-authors' email responses in one document and upload this file to your submission."
>
> Thank you,
>
> Rob
>
> UCLA HEALTH SCIENCES IMPORTANT WARNING: This email (and any attachments) is only intended for the use of the person or entity to which it is addressed, and may contain information that is privileged and confidential. You, the recipient, are obligated to maintain it in a safe, secure and confidential manner. Unauthorized redisclosure or failure to maintain confidentiality may subject you to federal and state penalties. If you are not the intended recipient, please immediately notify us by return email, and delete this message from your computer.

--
Jonathan Zhao
University of California, Los Angeles
Biology B.S. | Class of 2022
Contact | (408)896-4337

8. Sepehr Hamidi

Re: article approval

Hamidi, Sepehr <SHamidi@mednet.ucla.edu>
Sat 11/27/2021 1:58 PM
To: Rankin, Carl <CRankin@mednet.ucla.edu>

Dear Dr. Rankin,

I agree with the changes and the final version.

Thanks,
Sepehr Hamidi

From: Rankin, Carl <CRankin@mednet.ucla.edu>
Sent: Saturday, November 27, 2021 9:22 AM
To: ARTIN SOROOSH <asoroosh@ucla.edu>; Fang, Kai <KFang@mednet.ucla.edu>; jillmayhoffman@gmail.com <jillmayhoffman@gmail.com>; KaMan.Law@kp.org <KaMan.Law@kp.org>; Videlock, Elizabeth J. <EVidelock@mednet.ucla.edu>; ali.lok97@gmail.com <ali.lok97@gmail.com>; Jonathan Zhao <jonathan.zhao2000@gmail.com>; Hamidi, Sepehr <SHamidi@mednet.ucla.edu>; David Padua <davepadua@gmail.com>; Frey, Mark <mfrey@chla.usc.edu>; Harry Pothoulakis <hpothoulakis@yahoo.com>
Subject: article approval

Dear Authors,

One condition from the journal is that I compile a document containing email-based approvals from each author. The only difference between this version and the revision version is the addition of author contribution and data availability statements.

Here is their exact wording:

"Please request agreement from all authors including additions and deletions, these can be collected in the following way:

Email your co-authors with the change, and ask them to reply to your email confirming that they agree to these changes. Once you have collected these replies, please combine all of the co-authors' email responses in one document and upload this file to your submission."

Thank you,

Rob

9. David Padua

Re: article approval

David Padua <davepadua@gmail.com>
Sat 11/27/2021 12:12 PM
To: Rankin, Carl <CRankin@mednet.ucla.edu>

CAUTION - EXTERNAL EMAIL: Do not click links or open attachments unless you recognize the sender.

I approve

Sent from my iPhone

> On Nov 27, 2021, at 9:23 AM, Rankin, Carl <CRankin@mednet.ucla.edu> wrote:
>
> Dear Authors,
>
> One condition from the journal is that I compile a document containing email-based approvals from each author. The only difference between this version and the revision version is the addition of author contribution and data availability statements.
>
> Here is their exact wording:
>
> "Please request agreement from all authors including additions and deletions, these can be collected in the following way:
>
> Email your co-authors with the change, and ask them to reply to your email confirming that they agree to these changes. Once you have collected these replies, please combine all of the co-authors' email responses in one document and upload this file to your submission."
>
> Thank you,
>
> Rob
>
> UCLA HEALTH SCIENCES IMPORTANT WARNING: This email (and any attachments) is only intended for the use of the person or entity to which it is addressed, and may contain information that is privileged and confidential. You, the recipient, are obligated to maintain it in a safe, secure and confidential manner. Unauthorized redisclosure or failure to maintain confidentiality may subject you to federal and state penalties. If you are not the intended recipient, please immediately notify us by return email, and delete this message from your computer.
> <FINAL miR24.pdf>
> <Supplemental Material.pdf>
> <Revision Figures.pdf>

10. Mark Frey

Re: article approval

Frey, Mark <mfrey@chla.usc.edu>
Tue 11/30/2021 9:56 AM
To: Rankin, Carl <CRankin@mednet.ucla.edu>

1 attachments (225 KB)
FINAL miR24.pdf;

CAUTION - EXTERNAL EMAIL: Do not click links or open attachments unless you recognize the sender.

I agree with the changes!

Best
m

Mark R. Frey, Ph.D.
Associate Professor of Pediatrics and Biochemistry & Molecular Medicine
The Saban Research Institute at Children's Hospital Los Angeles
University of Southern California
4650 Sunset Blvd., Mailstop #137 | Los Angeles, CA 90027
Ph: 323.361.7204 | mfrey@usc.edu

> On Nov 30, 2021, at 9:28 AM, Rankin, Carl <CRankin@mednet.ucla.edu> wrote:
>
> **CAUTION: BE CAREFUL WITH THIS MESSAGE**
> This email came from outside CHLA. Do not open attachments, click on links, or respond unless you expected this message and recognize the email address: prvs=09684aa7a1=crankin@mednet.ucla.edu.
>
> Dear Artin, Harry, Jill and Mark
>
> I sent this to you over the weekend but included the figures so it was a really large email and Artin didn't get it. I wanted to send the manuscript alone to get your approval as the figures have not changed. See below remark from the journal.
>
> Thank you
>
> Rob
>
> **From:** Rankin, Carl <CRankin@mednet.ucla.edu>
> **Date:** Saturday, November 27, 2021 at 9:22 AM
> **To:** ARTIN SOROOSH <asoroosh@ucla.edu>, Fang, Kai <KFang@mednet.ucla.edu>, jillmayhoffman@gmail.com <jillmayhoffman@gmail.com>, KaMan.Law@kp.org <KaMan.Law@kp.org>, Videlock, Elizabeth J. <EVidelock@mednet.ucla.edu>, ali.lok97@gmail.com <ali.lok97@gmail.com>, Jonathan Zhao <jonathan.zhao2000@gmail.com>, Hamidi, Sepehr <SHamidi@mednet.ucla.edu>, David Padua <davepadua@gmail.com>, Frey, Mark <mfrey@chla.usc.edu>, Harry Pothoulakis <hpothoulakis@yahoo.com>
> **Subject:** article approval

11. Harry Pothoulakis

Gmail **Robert Rankin <rankincr@gmail.com>**

journal approval (rob rankin)

Harry Pothoulakis <hpothoulakis@yahoo.com> Wed, Dec 1, 2021 at 2:05 PM
To: Robert Rankin <rankincr@gmail.com>

Hi Rob: first time I see this e mail.
I have no problem with the change and I approve it.
Best, Harry P

Sent from my iPhone

> On Dec 1, 2021, at 4:46 PM, Robert Rankin <rankincr@gmail.com> wrote:
>
> [Quoted text hidden]

12. Corresponding author
