# Supplementary material for: Loss of miR-24-3p promotes epithelial cell apoptosis and impairs the recovery from intestinal inflammation
Source: Cell Death Dis. 2021 Dec 18;13(1):8. doi: 10.1038/s41419-021-04463-4 (PMC8684555; doi:10.1038/s41419-021-04463-4)
Supplement: Supplementary file 3 — Supplemental Figures and Legends [file 41419_2021_4463_MOESM3_ESM.pdf]

Supplemental Figure 1

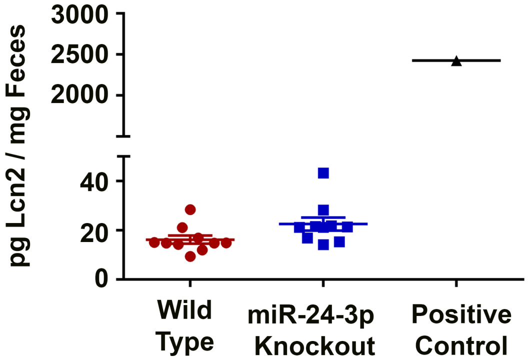

Supplemental Figure 2

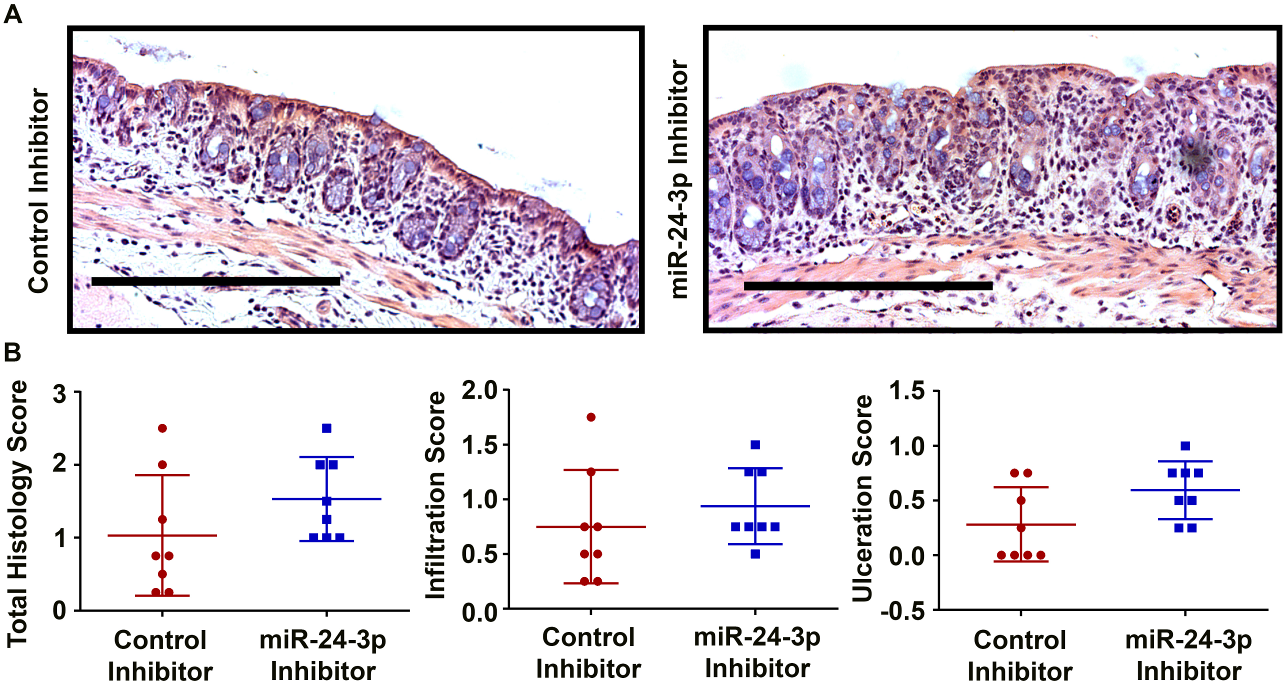

Supplemental Figure 3

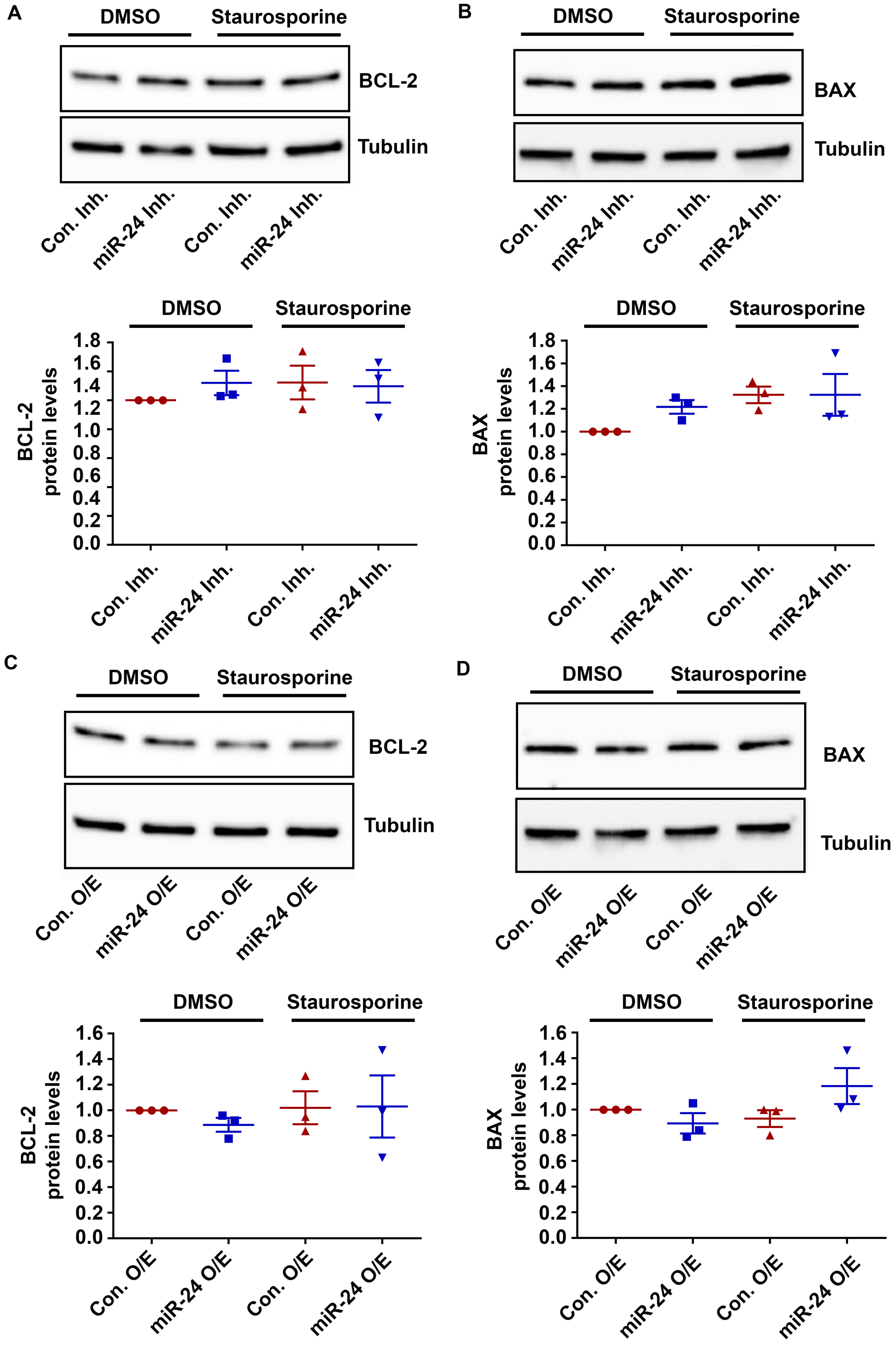

Supplemental Figure Legends

Supplemental Figure 1: miR-24-3p knockout mice do not display spontaneous colitis. After collection of feces from both male and female adult mice a Lipocalin-2 ELISA was performed. The positive control is feces from a wild-type mouse with acute DSS colitis. n = 10 mice/group. Mean ± SD.

Supplemental Figure 2: miR-24-3p inhibition does not affect the onset of DSS colitis. (A) Representative H&E stained swiss rolled colons from day 5 control inhibitor and miR-24-3p inhibitor treated mice. Scale bars = 0.25 mm. (B) The average total, infiltrate, and ulceration scores from 2 double blinded reviewers of day 5 inhibitor treated mice. Mean ± SD. n = 8 mice / group.

Supplemental Figure 3: miR-24-3p overexpression or inhibition does not influence the protein levels of BCL-2 or BAX. (A,B) Western blots and densitometric analysis of BCL-2 (A) and BAX (B) after overnight treatment with inhibitors and a 4 hr treatment with staurosporine. 3 independent experiments. Mean ± SEM. (C,D) Western blots and densitometric analysis of BCL-2 (A) and BAX (B) after overnight treatment with miRNA mimetics and a 4 hr treatment with staurosporine. 3 independent experiments. Mean ± SEM.
